# *In-Vitro* Suppression of IL-6 and IL-8 Release from Human Pulmonary Epithelial Cells by Non-Anticoagulant Fraction of Enoxaparin

**DOI:** 10.1371/journal.pone.0126763

**Published:** 2015-05-11

**Authors:** Madhur D. Shastri, Niall Stewart, James Horne, Gregory M. Peterson, Nuri Gueven, Sukhwinder S. Sohal, Rahul P. Patel

**Affiliations:** 1 Pharmacy, School of Medicine, Faculty of Health, University of Tasmania, Hobart, Tasmania, Australia; 2 Central Science Laboratory, University of Tasmania, Hobart, Tasmania, Australia; 3 Health Services Innovation Tasmania, School of Medicine, Faculty of Health, University of Tasmania, Hobart, Tasmania, Australia; 4 Breathe Well Centre of Research Excellence for Chronic Respiratory Disease and Lung Ageing, School of Medicine, Faculty of Health, University of Tasmania, Hobart, Tasmania, Australia; 5 School of Health Sciences, Faculty of Health, University of Tasmania, Launceston, Tasmania, Australia; University of Patras, GREECE

## Abstract

**Background:**

Enoxaparin, a mixture of anticoagulant and non-anticoagulant fractions, is widely used as an anticoagulant agent. However, it is also reported to possess anti-inflammatory properties. Our study indicated that enoxaparin inhibits the release of IL-6 and IL-8 from A549 pulmonary epithelial cells. Their release causes extensive lung tissue damage. The use of enoxaparin as an anti-inflammatory agent is hampered due to the risk of bleeding associated with its anticoagulant fractions. Therefore, we aimed to identify the fraction responsible for the observed anti-inflammatory effect of enoxaparin and to determine the relationship between its structure and biological activities.

**Methods:**

A549 pulmonary epithelial cells were pre-treated in the presence of enoxaparin and its fractions. The levels of IL-6 and IL-8 released from the trypsin-stimulated cells were measured by ELISA. The anticoagulant activity of the fraction responsible for the effect of enoxaparin was determined using an anti-factor-Xa assay. The fraction was structurally characterised using nuclear magnetic resonance. The fraction was 2*-O*, 6-*O* or *N*-desulfated to determine the position of sulfate groups required for the inhibition of interleukins. High-performance size-exclusion chromatography was performed to rule out that the observed effect was due to the interaction between the fraction and trypsin or interleukins.

**Results:**

Enoxaparin (60μg/mL) inhibited the release of IL-6 and IL-8 by >30%. The fraction responsible for this effect of enoxaparin was found to be a disaccharide composed of α-L-iduronic-acid and α-D-glucosamine-6-sulfate. It (15μg/mL) inhibited the release of interleukins by >70%. The 6-*O* sulphate groups were responsible for its anti-inflammatory effect. The fraction did not bind to trypsin or interleukins, suggesting the effect was not due to an artefact of the experimental model.

**Conclusion:**

The identified disaccharide has no anticoagulant activity and therefore eliminates the risk of bleeding associated with enoxaparin. Future *in-vivo* studies should be designed to validate findings of the current study.

## Introduction

Asthma, a respiratory inflammatory disorder, affects millions of people around the world and its prevalence has increased markedly in recent years [1]. The pathogenesis of asthma is complex, involving different cell types, such as T-cells, neutrophils, mast cells, eosinophils, macrophages and epithelial cells of the lung [2]. Epithelial cells and macrophages act as the first-line of defence before the migration and recruitment of other types of inflammatory cells into the lungs upon exposure to various foreign particles, including allergens [3,4]. Among various pro-inflammatory mediators, interleukin (IL)-6 and IL-8 are released by the pulmonary epithelial cells in response to their damage or stress caused by a variety of foreign particles [5]. IL-6 is a pro-inflammatory cytokine and its involvement in various immuno-regulatory effects, such as increased IL-4 production during Th2 differentiation, increased Th17 differentiation, decreased Th1 differentiation and increased IL-4 dependent synthesis of immunoglobulins, causes further damage to the lungs [6,7]. IL-8, a potent chemotactic agent, facilitates the migration of neutrophils and T-cells, and priming of eosinophils [8]. These immune cells can then cause extensive tissue damage and prolong the inflammatory phase.

Corticosteroids are currently used as first line agents for the treatment of asthma and are effective in supressing the release of various inflammatory mediators involved in the pathogenesis of disease. However, they are associated with severe side effects, especially with their long-term use in children, and there has been a consistent rise in the prevalence of difficult to treat corticosteroid-resistant asthma [9,10]. More recently, monoclonal antibodies have been developed to treat various immunological disorders, including asthma. For example, omalizumab, a humanised monoclonal-immunoglobulin specific antibody, is the only biological approved for the treatment of severe, uncontrolled and steroid-resistant asthma. However, apart from its high cost and inconvenient route of administration, it is associated with serious side effects, such as anaphylactic reactions as well as occurrence of cardiovascular and cerebrovascular adverse effects [11,12]. Therefore, there is a need for the development of novel therapeutic modalities for management of asthma.

Enoxaparin, a well-known anticoagulant drug, has recently attracted much research interest for its anti-inflammatory properties. This low-molecular-weight heparin (LMWH) belongs to the family of glycosaminoglycans and is composed of various fractions, also known as oligosaccharides, of unfractionated heparin (UFH) [13]. Although enoxaparin is commonly used for the prophylaxis of deep vein thrombosis [14], clinical studies have implicated its usefulness for the management of various inflammatory conditions, including asthma [15–17]. Like UFH and other LMWHs, enoxaparin is also composed of a mixture of anticoagulant and non-anticoagulant oligosaccharides [18]. Unfortunately, given the increased risk of bleeding associated with anticoagulant oligosaccharides of enoxaparin, its use is not indicated for the management of inflammatory disorders [19]. However, clinical studies have indirectly indicated that the observed anti-inflammatory effects of heparins (UFH and LMWHs) are because of the presence of non-anticoagulant oligosaccharides and, hence, independent of their anticoagulant effect [20,21]. One potential way to avoid the bleeding complications associated with heparins when used for the management of inflammatory conditions is to identify the non-anticoagulant oligosaccharides responsible for their anti-inflammatory effects. The current approach to obtain the oligosaccharides is to perform depolymerisation of heparins by chemical or enzymatic methods. However, a chemical or enzymatic depolymerisation results in the structural modification of oligosaccharides and it has been demonstrated that certain biological functions of the parent heparin could indeed be removed by the depolymerisation process [22–24]. Some oligosaccharides in heparins are heat sensitive and can undergo chemical modification, especially desulfation, with the elevated temperatures of the depolymerisation process [25]. An oligosaccharide’s sulfation pattern is a key determinant for its anti-inflammatory properties. Depolymerisation can also be performed through a freeze-drying process; however, freeze-drying results in physical changes of some oligosaccharides within the heparin molecule [26].

An alternative approach is to separate, isolate and identify the non-anticoagulant oligosaccharides of heparins without their prior chemical or enzymatic digestion. However, a major limitation in the separation of heparins is the lack of a high resolution technique as they are highly negatively charged and structurally complicated compounds [27,28]. Nevertheless, we have recently developed a novel ion-exchange chromatographic (IC) technique that can effectively separate the anticoagulant and non-anticoagulant oligosaccharides of enoxaparin without their structural modification [29]. Since our study indicated that enoxaparin can inhibit the release of two key pro-inflammatory mediators involved in the pathogenesis of asthma (IL-6 and IL-8), we first identified the IC-derived fraction responsible for the observed inhibitory effect of enoxaparin and then characterised its structure, as well as determined the specific position of sulfate groups required for the inhibition of IL-6 and IL-8 release.

## Materials and Methods

### Reagents

A549 human pulmonary epithelial cell line was purchased from the American Type Culture Collection (Manassas, VA, USA). Enoxaparin was obtained from Aventis Pharma Ltd. (NSW, Australia). UFH was purchased from Hospira Pty. Ltd. (Victoria, Australia). Sodium chloride, tetrahydrofuran, sodium hydroxide, *N*-methyl-*N*-(trimethylsilyl) trifluroacetamide, methanol, acetic acid, Ham’s F12K medium, antibiotics (penicillin G and streptomycin), trypsin-ethylenediaminetetraacetic acid (EDTA), trypsin, thrombin, enzyme-linked immunosorbent assay (ELISA) kits for IL-6 and IL-8, trypan blue exclusion assay kit, lactate dehydrogenase (LDH) activity assay kit, and potassium phosphate monobasic and dibasic were purchased from Sigma-Aldrich (Castle Hill, NSW, Australia). Fetal bovine serum, IL-6 and IL-8 recombinant human proteins were obtained from Invitrogen (Grand Island, NY, USA). Deuterium oxide (D_2_O) was purchased from Cambridge Isotope Laboratories (Andover, MA, USA).

### Human Pulmonary Epithelial Cell (A549) Culture

For initial growth, the A549 cells were cultured in 75 cm^2^ tissue culture flasks (Corning, NL, Mexico) and grown to confluence in complete medium [Ham’s F-12 Kaighn’s Modification medium supplemented with 10% fetal bovine serum, 2 mM L-glutamine and 100 μg/mL antibiotics (penicillin G and streptomycin)] and incubated at 37°C in a humidified 5% CO_2_ atmosphere as described previously [30].

### Preparation of Stock Solutions

Stock solutions of enoxaparin at 1 mg/mL were prepared in serum free medium (Ham’s F12K) and filter sterilized through 0.2 μm pore size syringe filters (Pall Life Sciences, Victoria, Australia). Other stock solutions were prepared accordingly: 1.0 μg/mL trypsin (A549 human pulmonary epithelial cell stimulant) in Ham’s F12K, stored at -20°C.

### Preparation of Epithelial Cell Culture Supernatants

At confluence, the cells were detached from the flasks using trypsin-EDTA solution and re-seeded in 24-well cell culture plates at a density of 1 × 10^5^ cells/well. The cells were incubated for 24 hours and grown to confluency in serum free medium. After reaching confluency, the cells were exposed to 0.003 μg/mL of trypsin in the presence of either Ham’s F12K medium (negative control) or different concentrations (5, 10, 20, 40, 60, 80 and 100 μg/mL) of enoxaparin. After 24 hours of incubation (37°C, humidified 5% CO_2_ atmosphere), cultures were centrifuged, and supernatants were removed and analysed for the levels of IL-6 and IL-8 using ELISA.

### Analysis of IL-6 and IL-8 in Epithelial Cell Supernatants

ELISA kits specific for the measurement of human IL-6 and IL-8 (pre-coated with capture antibody) were prepared as per manufacturer’s recommendations. Briefly, a known volume of standards and culture supernatants (100 μL and 50 μL for IL-6 and IL-8, respectively) was added to the pre-coated wells. Further, 50 μL/well of biotinylated detection antibody was added to each plate and incubated for 2 hours at room temperature and washed 5 times. Next, 100 μL/well of standard horseradish peroxidase conjugated streptavidin (a commonly used enzyme to modify substrate resulting in colour development) was added to each well and incubated for 30 minutes at room temperature in the dark. Plates were again washed 5 times and 100 μL/well of stabilised chromogen was added to each well. Plates were then allowed to stand in the dark for 30 minutes and the reaction was quenched using 100 μL/well of stop solution (1N hydrochloric acid). Measurement of the optical density was performed using a plate reader (Spectra Max M2 microplate reader, Sunnyvale, CA) at 450 nm. Each epithelial cell treatment was performed in triplicate and supernatants of each treatment were analysed in duplicate.

### Viability and Cytotoxicity Studies of Enoxaparin on Epithelial Cells

The effect of enoxaparin at maximal inhibitory concentration on either cellular viability of A549 cells or its cytotoxic effect on cells after 24 hours of incubation was assessed using two routinely used methods. The viability of cells was determined by the trypan blue exclusion. The cytotoxicity of enoxaparin treatment was determined using the LDH *in-vitro* toxicology assay, as described before [31]. Briefly, the culture supernatants were centrifuged at 250g for 4 minutes. An aliquot of either blank (complete medium) or control (epithelial cells only) cells treated with trypsin or co-incubated with enoxaparin, was mixed with an aliquot of solution containing LDH assay mixture (LDH substrate, LDH dye and LDH cofactor). The mixture was incubated at room temperature for 20–30 minutes and the reaction was quenched by the addition of 1 N hydrochloric acid (15 μL). The absorbance was measured spectrophotometrically using a plate reader (Spectra Max M2 microplate reader, Sunnyvale, CA) at a wavelength of 490 nm. Each sample was prepared and analysed in triplicate.

### Separation of Enoxaparin

The chromatographic separation of enoxaparin was carried out using a previously described ion-exchange chromatography (IC) technique [29] with minor modifications. Separations were performed on a biocompatible chromatography system (Thermo Fisher Scientific, NSW, Australia) using a semi-preparative Dionex CarboPac PA100 (250 mm, 9 mm ID, 8.5 μm) strong anion-exchange column (Thermo Fisher Scientific, NSW, Australia). The chromatography system consisted of a HPG-3400RS binary separation pump, WPD-3000RS auto-sampler, TCC-3000RS column thermal compartment and UV-3000RS detector. The column thermal compartment was set at a temperature of 40°C and UV detection was performed at 232 nm. An injection volume of 250 μL was chosen for this work, which allowed 10 mg of enoxaparin to be loaded onto the column for each injection, and a total flow rate of 2.0 mL/minute was maintained throughout. Instrument control and data acquisition was performed using Chromeleon software. The mobile phases were composed of Milli-Q water (A) and 2 M NaCl (B). The 2 M NaCl stock solution was prepared by mixing NaCl and Milli-Q water and the solution was filtered and degassed off-line. The optimised NaCl eluent gradient (mobile phase B) was from 32 to 74% over 0 to 70 minutes. The 14 fractions of enoxaparin were collected in centrifuge tubes over the elution period between 29 to 65 minutes. The collected fractions containing NaCl (0.64–1.48 M) were then subjected to desalting procedures.

### Desalting of Enoxaparin Fractions

The IC-derived fractions of enoxaparin were desalted as described previously [29] with minor modifications. Each fraction was concentrated on a miVac DNA centrifugal concentrator (Genevac Ltd, Suffolk, UK) at 40°C. The concentrated solutions containing NaCl and enoxaparin fractions were desalted using PD MidiTrap G-10 columns (GE Healthcare Life Sciences, Uppsala, Sweden) having desalting capacity of more than 95%. The recovery of each fraction was determined by reanalysing the desalted fractions using IC under the same chromatographic conditions as described above. The concentration of each fraction was calculated using the differences in the peak areas of the desalted fraction and enoxaparin fraction eluted at the same time.

The stock solution (1 mg/mL) of each fraction was prepared in serum-free Ham’s F12K medium. The fractions were tested for their effects on the release of IL-6 and IL-8 from stimulated human pulmonary epithelial cells, as described above. Anticoagulant activity of the fraction responsible for the inhibitory effect of enoxaparin was determined using an anti-factor Xa assay. Its structural characterisation was then carried out using a nuclear magnetic resonance (NMR) technique.

### NMR Analysis of Enoxaparin Fraction

The identified fraction was dissolved in potassium phosphate buffer at pH 7.0 prepared in 99.9% D_2_O. All data were acquired with a Bruker AVANCE III HD 4-channel spectrometer with a 5mm triple resonance cryogenically cooled probe. Sample temperature was regulated at 298K and spectrometer field frequency was 600.07 MHz and 150.88 MHz for ^1^H and ^13^C nuclei, respectively. Data acquisition and processing were executed within Topspin 3.2 software (Bruker Corporation, MA, USA). All chemical shifts are referenced indirectly to DSS (NMR standard) at 0.0 ppm in ^1^H and ^13^C. The experiments conducted and their relevant acquisition and processing details are as follows: ^1^H-1D (“noesgppr1d”) with presaturation of residual solvent resonance, acquired with 64K datapoints in 8 transients, spectral width of 10 ppm and a relaxation delay of 4 seconds. Raw data was processed with an exponential apodisation with a line-broadening factor of 0.3 Hz prior to Fourier transformation; ^13^C-1D (“zgpg30”) with proton decoupling acquired with 64K datapoints in 20,000 transients, spectral width of 200 ppm and relaxation delay of 2 seconds. Raw data was processed with an exponential apodisation with a line-broadening factor of 1.0 Hz prior to Fourier transformation; ^1^H-^13^C-HSQC (“hsqcedetgpsisp2.2”) with multiplicity editing acquired with 2048 × 64 datapoints, 2 transients per 2D increment, spectral widths of 10 ppm and 165 ppm for ^1^H and ^13^C dimensions, respectively, and a relaxation delay of 1.5 seconds. Data were processed with zero filling in both dimensions and a squared sine apodisation (SSB = 2) to a resultant matrix of 4096 × 1024 datapoints; ^1^H-^13^C-HMBC (“hmbcgpl2ndqf”) acquired with 2048 × 128 datapoints, 16 transients per 2D increment, spectral widths of 10 ppm and 200 ppm for ^1^H and ^13^C dimensions, respectively, and a relaxation delay of 1.5 seconds. Data were processed with zero filling in both dimensions and a squared sine apodisation (SSB = 2) to a resultant matrix of 4096 × 1024 datapoints; ^1^H-^13^C-HSQCTOCSY (“hsqcetgpml”) acquired with 2048 × 256 datapoints, 32 transients per 2D increment, spectral widths of 10 ppm and 165 ppm for ^1^H and ^13^C dimensions, respectively, TOCSY mixing time of 120 milliseconds and a relaxation delay of 1.5 seconds. Data were processed with zero filling in both dimensions and a squared sine apodisation (SSB = 2) to a resultant matrix of 4096 × 1024 datapoints.

### Desulfation of Enoxaparin Fraction

#### Selective 2-O desulfation

Selective 2-*O*-desulfation of identified fraction was performed using a previously described method [32]. Briefly, a solution containing 8 mg/mL of fraction was dissolved in 200 μL of 0.1 M sodium hydroxide. The mixture was then frozen and lyophilised. The residues were dissolved in 0.5 mL of Milli-Q water and acetic acid was added to adjust the pH of the mixture to 8.0. The mixture was evaporated to dryness and precipitated using 80% *v/v* of anhydrous methanol, followed by centrifugation at 3000 rpm for 10 minutes. The supernatant was carefully discarded and samples were kept at 4°C overnight. Any traces of methanol were removed using a miVac DNA centrifugal concentrator and the remaining precipitants were dissolved in 4 mL Milli-Q water to obtain 2-*O*-desulfated fraction of enoxaparin.

#### N-desulfation

A solution containing 8 mg/mL of enoxaparin fraction was mixed with 650 μL of tetrahydrofuran and 50 μL of Milli-Q water and incubated for 30 minutes at 50°C for partial *N*-desulfation, as described previously [32] with minor modifications. The solution was neutralised using 0.1 M sodium hydroxide. The resulting mixture was evaporated to dryness and precipitated by the addition of anhydrous methanol as described above and dissolved in 4 mL Milli-Q water.

#### Selective 6-O-desulfation

Selective 6-*O*-desulfation of enoxaparin fraction was performed as previously described [32]. Briefly, 8 mg/mL of the fraction was mixed with 1 mL of solvent (tetrahydrofuran) and 1 mL of silylating agent (*N*-methyl-*N*-(trimethylsilyl) trifluroacetamide). The solution was incubated for 9 hours at 50°C. The resulting mixture was evaporated to dryness and precipitated by the addition of anhydrous methanol as described above and the precipitants were dissolved in 4 mL Milli-Q water.

After desulfation, free sulfate groups were removed using a 600Da cut-off filter (Millipore, NSW, Australia) at 15000 rpm for 10 minutes. The concentrated supernatant was dissolved in 1 mL of Milli-Q water for further use. Each selectively desulfated enoxaparin sample was tested for its effect on the release of IL-6 and IL-8 from stimulated human pulmonary epithelial cells. Each sample was prepared and analysed in triplicate.

### Binding of Proteins to the Identified Fraction

#### High-Performance Size Exclusion Chromatography (HP-SEC) Instrumentation

The putative binding of the identified fraction to either trypsin or ILs was determined by HP-SEC. The HP-SEC system (Thermo Fisher Scientific, NSW, Australia) consisted of a HPG-3400RS binary separation pump and a WPS-3000TRS auto-sampler. The system was connected to Corona Ultra RS charged aerosol detector (C-CAD; Thermo Fisher Scientific, NSW, Australia). C-CAD was used as per the manufacturer’s recommended settings of 35±0.2 psi for the nitrogen gas flow at 30°C nebuliser temperature. An injection volume of 10 μL and a total flow rate of 1.0 mL/minute were maintained. Instrument control and data acquisition were performed using Chromeleon software.

#### HP-SEC Analysis

HP-SEC analysis was performed using two HP-SEC columns coupled in series; a Superdex peptide 10/300 GL (300 × 10 mm) and a Superdex 75 10/300 GL (300 × 10 mm) column (GE Healthcare Life Sciences, Uppsala, Sweden) were used for this purpose. Isocratic elution of the tested analytes was performed with a mobile phase containing 100 mM ammonium acetate (pH 6.0) as eluent. The method was validated by investigating the intra- (n = 6) and inter-day (five consecutive days; n = 30) precision using peak areas of 10 μM of UFH, thrombin, trypsin, IL-6 or IL-8 or 100 μM of identified fraction. Mean intra- and inter-day accuracy was also determined and calculated as (observed concentration-expected concentration)/expected concentration × 100). Mean inter-day peak retention time for each of the analytes was also determined. The peak area and retention time of the analytes were determined using Cobra integration wizard software.

#### Sample Preparation for HP-SEC analysis

A stock solution containing 20μM of UFH, thrombin, trypsin, IL-6 or IL-8 or 1000 μM of identified fraction was prepared in potassium phosphate buffer. The stock solutions of thrombin, trypsin, IL-6 or IL-8 was diluted in ammonium acetate to obtain the concentration of 10 μM. The stock solution of UFH or identified fraction was diluted in Milli-Q water to obtain the concentration of 10 or 500 μM. Each sample was prepared and analysed in triplicate.

### Statistical Analysis

Data are presented as mean ± standard deviation (SD) or as percentage change in the release of IL-6 and IL-8 following different types of treatments compared to trypsin-stimulated controls. Statistical analysis was performed using GraphPad Prism (version 6, GraphPad Software Inc, CA, USA), and significance was evaluated using independent sample or paired Student’s *t*-test, and one way analysis of variance (ANOVA), where applicable, followed by Dunnett’s multiple comparison test. A *p*-value of <0.05 was considered statistically significant.

## Results and Discussion

### Release of IL-6 and IL-8

**Fig 1:**
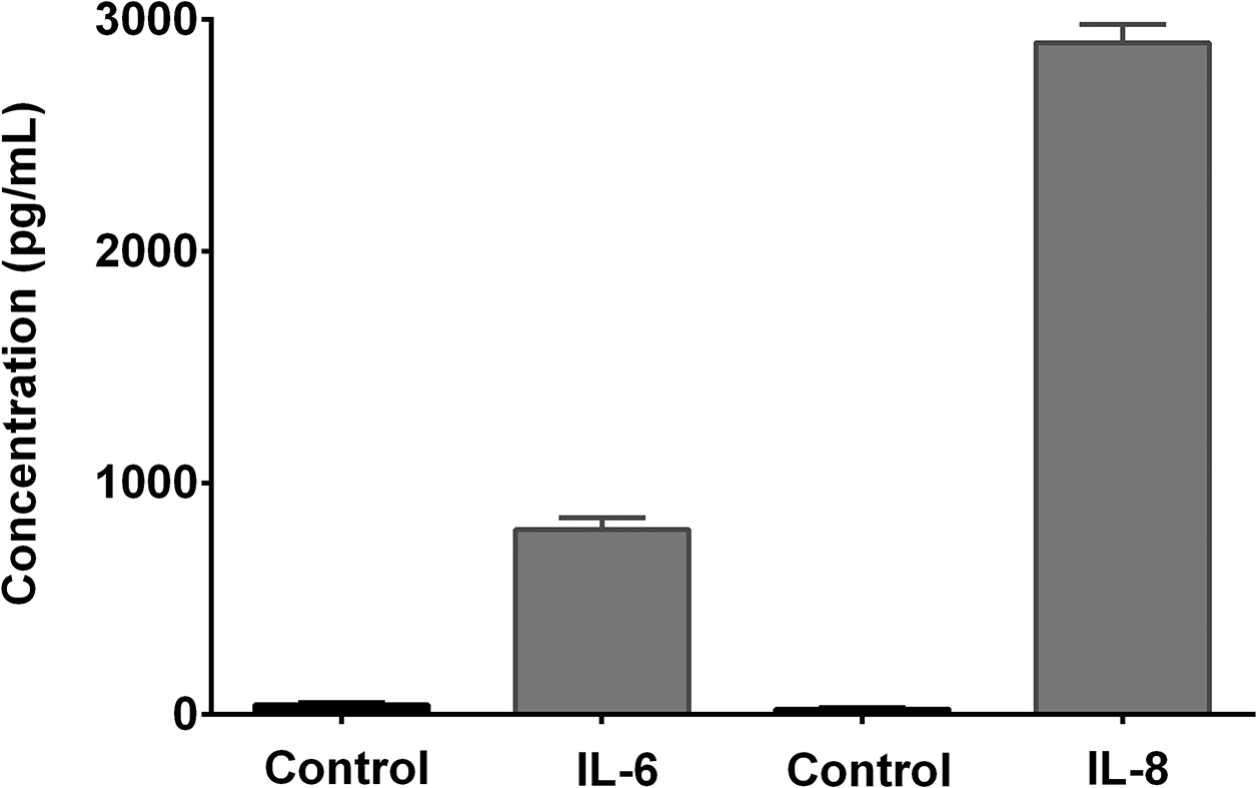
*In-vitro* interleukin release. Trypsin-induced release of IL-6 and IL-8 from the epithelial cell culture supernatants. Data is presented as mean ± SD.

The calibration curves used for the measurement of IL-6 and IL-8 were generated using seven recommended concentrations of respective IL standards. The linearity, estimated by correlation coefficient (r^2^), was greater than 0.988. The levels (pg/mL) of IL-6 and IL-8 measured 24 hours after trypsin-induced stimulation of A549 epithelial cells are shown in Fig 1. The baseline levels of IL-6 and IL-8 were 42 and 26 pg/mL, respectively. In the presence of trypsin, the levels of IL-6 and IL-8 were increased to 800 pg/mL and 2900 pg/mL (*p*<0.0001). In the current study, trypsin was used to activate the lung epithelial cells because it can induce the stimulation of proteinase-activated receptors (PAR) expressed by human alveolar as well as bronchial epithelial cell lines [33]. Recent *in-vivo* studies have shown that endogenous trypsin (mainly located in the lung) induced activation of PAR in human lung epithelial cells releases IL-6 and IL-8, resulting in the progressive loss of lung functions [34].

### Effect of Enoxaparin on IL-6 and IL-8 release

**Fig 2:**
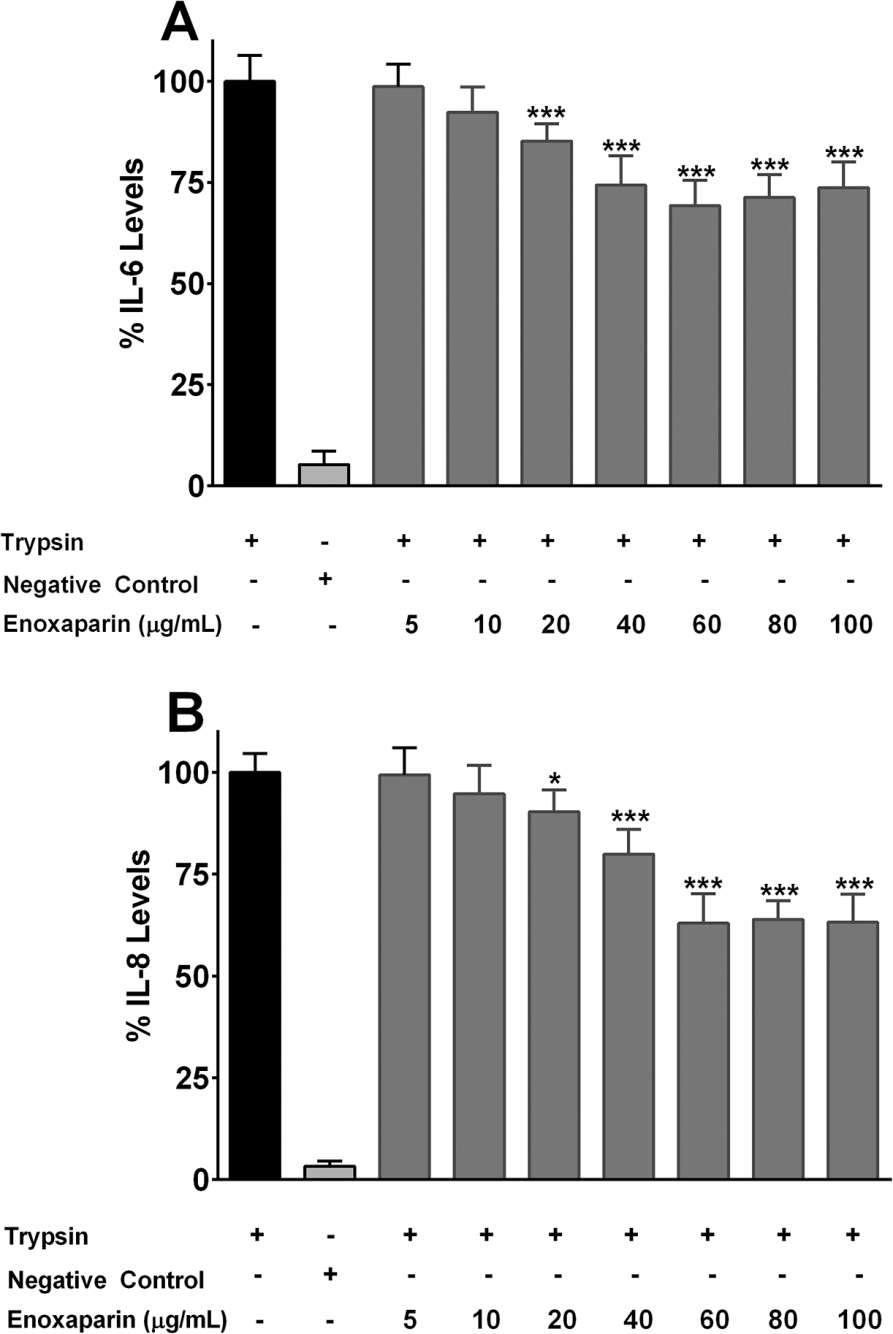
Concentration-dependent effect of enoxaparin on interleukin release. Effect of different concentration of enoxaparin on the release of IL-6 **(A)** and IL-8 **(B)** following trypsin-induced *in-vitro* stimulation epithelial cells. Data is presented as percentage (mean ± SD) of the maximal observed IL-6 and IL-8 concentrations. *p<0.05, and ***p<0.001 versus trypsin-stimulated control.

As IL-6 and IL-8 are likely to be a potential target for new treatment modalities for this important disease, their release from the lung epithelial cells was determined in the presence or absence of enoxaparin. It inhibited the release of IL-6 and IL-8 in a concentration dependent manner (Fig 2A and 2B). Compared to control samples, the levels of tested ILs were not statistically different in the presence of 5 and 10 μg/mL of enoxaparin (*p*>0.1466). The maximum inhibition was observed at 60 μg/mL where the release of IL-6 and IL-8 was inhibited by 31% and 37%, respectively (*p*<0.0001). A further increase in the concentration of intact enoxaparin did not result in greater inhibition of IL release. LMWHs have been reported to inhibit the release of various inflammatory cytokines, including IL-4, IL-5, IL-13 and tumour necrosis factor-alpha [35–37]. However, the current study for the first time demonstrated that enoxaparin can significantly inhibit the release of IL-6 and IL-8 from the stimulated lung epithelial cells.

### Effect of Enoxaparin on Cell Viability and Proliferation

**Fig 3:**
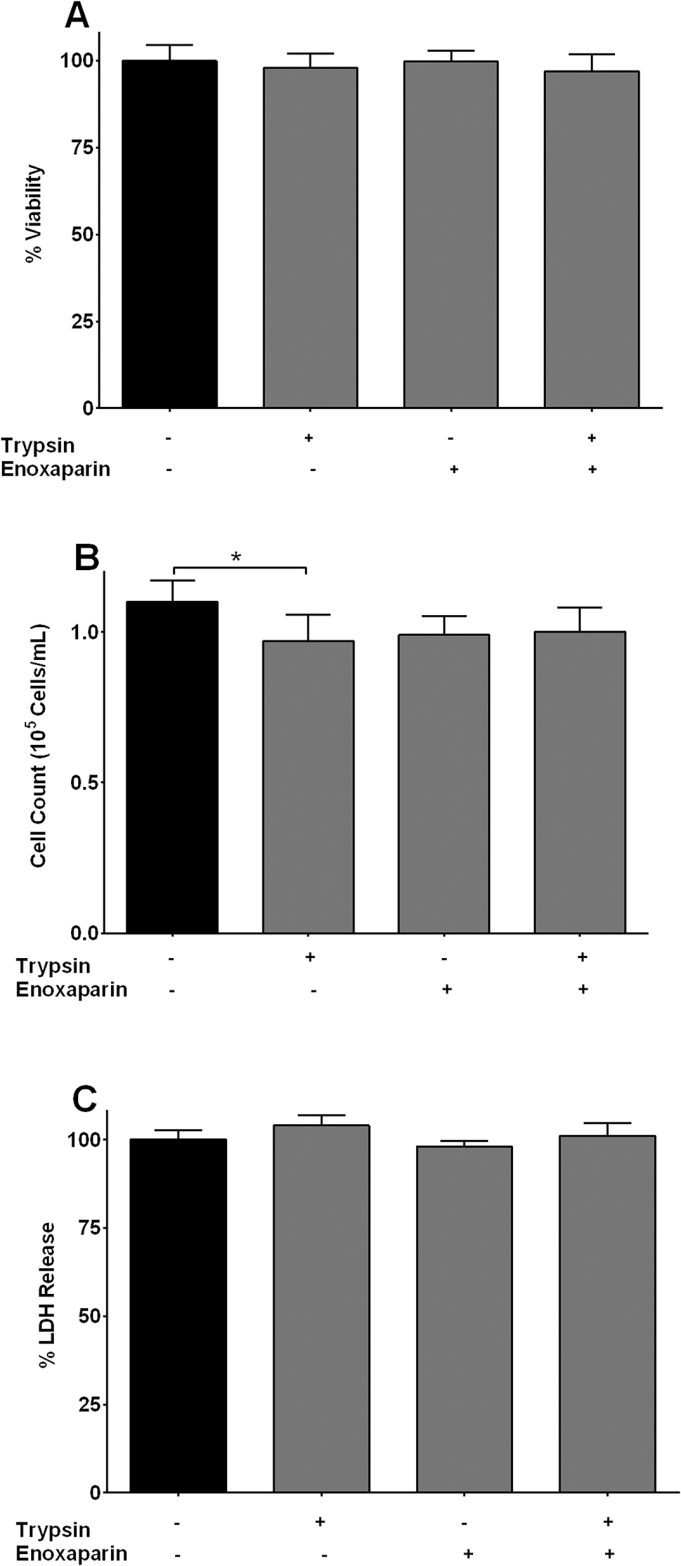
Effect of enoxaparin on cellular viability and proliferation. **(A)** Viability of epithelial cells was determined by trypan blue dye exclusion test and is presented as % of viable cells remaining after 24 hours of incubation with trypsin, enoxaparin or trypsin + enoxaparin. Data is presented as mean ± SD. **(B)** Proliferation of epithelial cells was determined after 24 hours in the presence of trypsin, enoxaparin or trypsin + enoxaparin. Cells were counted after incubation for 24 hours and expressed as 10^5^ cells/mL. Data is presented as mean ± SD. *p<0.05 versus unstimulated control. **(C)** Viability of epithelial cells is presented as mean % LDH release ± SD. Cells were incubated for 24 hours in the presence of trypsin, enoxaparin or trypsin + enoxaparin.

To rule out that the observed inhibitory effect was because of enoxaparin-induced cytotoxicity or inhibition of cell proliferation, the cell viability and proliferation assays were performed in the presence or absence of intact enoxaparin. The concentration of 60 μg/mL of intact enoxaparin was selected to determine its effect on the viability and proliferation of A549 human pulmonary epithelial cells. After 24 hours of incubation, 60 μg/mL of enoxaparin did not reduce the viability of epithelial cells compared to the control. Similarly, no effect on the number of cells (proliferation) was observed after the addition of 60 μg/mL of enoxaparin compared to the control (Fig 3A and 3B). Additionally, enoxaparin did not induce cellular toxicity since it was not found to increase the release of LDH in epithelial cell culture supernatants (Fig 3C). These results indicated that the suppression of IL-6 or IL-8 release by enoxaparin was not related to its cytotoxic effect or changes in cellular proliferation.

### Effect of Enoxaparin fractions on IL-6 and IL-8 Release

**Fig 4:**
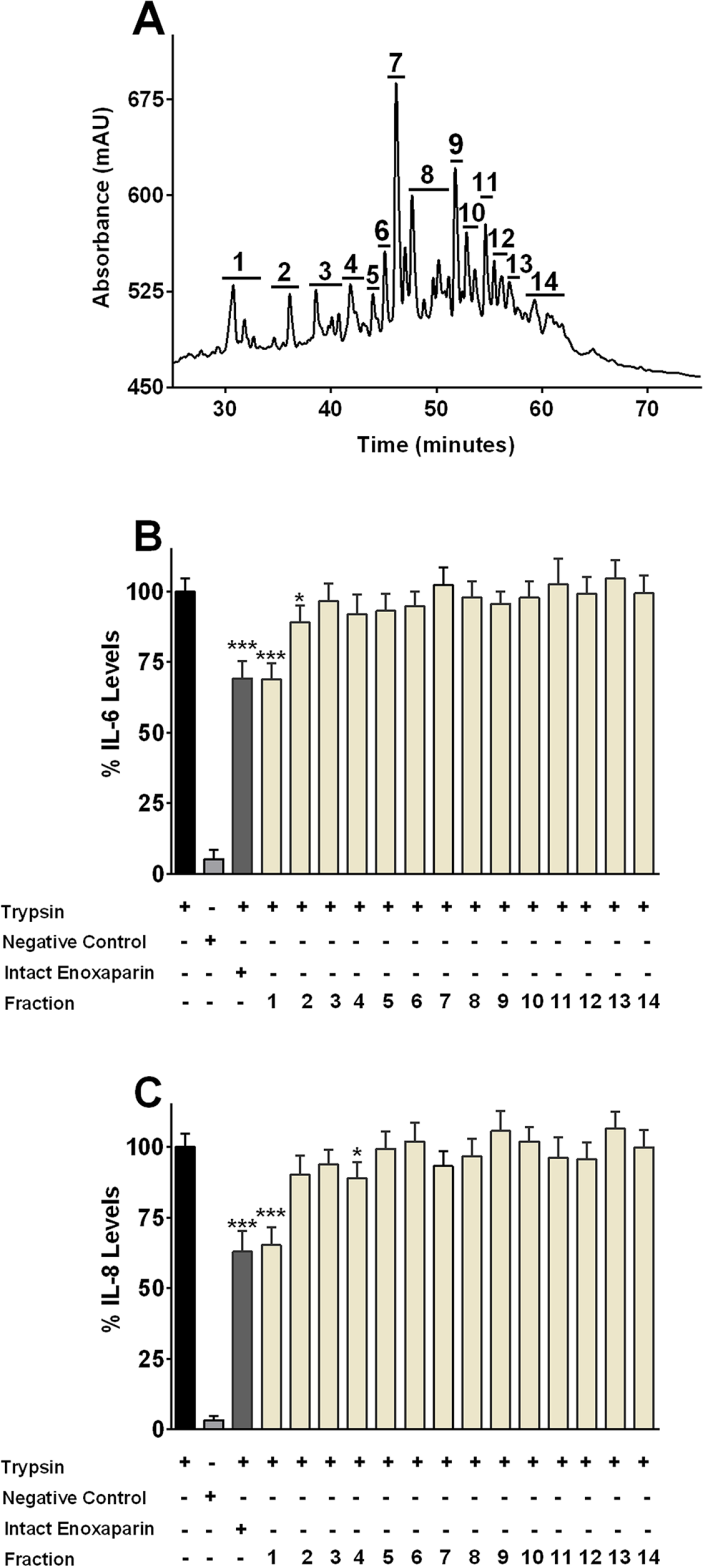
Effect of enoxaparin fractions on interleukin release. Ion-exchange chromatographic (IC) separation of enoxaparin. The separations were performed using a CarboPac PA100 semi-preparative column; optimised NaCl gradient was from 32–74% over 70 minutes with a flow rate of 2 mL/minute and UV detection wavelength of 232 nm. The numbers indicate the area of all the fractions collected. Data represents a typical experiment out of ten independent experiments **(A)**. Inhibition of IL-6 **(B)** and IL-8 **(C)** release by IC-derived 14 fractions of enoxaparin (60 μg/mL) after trypsin-induced *in-vitro* stimulation of epithelial cells. The relative percentile amount of each fraction (fraction 1 to 14) present in 60 μg/mL of intact enoxaparin was: 9.18%, 4.24%, 6.12%, 5.15%, 2.99%, 4.78%, 20.32%, 11.4%, 10.53%, 7.35%, 5.98%, 4.6%, 2.95% and 5.06% respectively. Data is presented as percentage (mean ± SD) of the maximal observed IL-6 and IL-8 concentrations. *p<0.05, and ***p<0.001 versus trypsin-stimulated control.

Enoxaparin is a mixture of structurally complicated and unidentified fractions. It has been suggested that different fractions of enoxaparin have different biological activities. For example, the pentasaccharide sequence (5 saccharides) of enoxaparin is required for its anticoagulant effect [38]. On the other hand, hexasaccharides (6 saccharides) of enoxaparin are effective in inhibiting the macrophage-induced release of nitric oxide [29]. Therefore, to find out the fraction(s) responsible for the observed inhibitory effect of the parent compound, enoxaparin was separated into different fractions using an IC technique (Fig 4A). The effects of 14 IC-derived fractions on the release of IL-6 and IL-8 are shown in Fig 4B and 4C. The 14 fractions were selected based on their previously reported molecular weights [29]. For example, the molecular weight of fraction 1, fractions 2, 3 and 4, and fraction 5, 6 and 7 were found to be approximately 600, 1200 and 1800 Da, respectively. The tested concentration of each fraction reflected their concentration present in 60 μg/mL of enoxaparin (at which enoxaparin exhibited the maximal level of IL-6 and IL-8 inhibition).

Various fractions inhibited the release of IL-6 and IL-8 to different extents. While the majority of fractions did not significantly change the levels of IL-6 and IL-8 (*p*>0.0614), fraction 1 inhibited the release of IL-6 and IL-8 release by 30% and 35%, respectively (*p*<0.0001), and this effect was comparable to the suppression displayed by enoxaparin. Fraction 2 and 4 were found to inhibit the release of IL-6 and IL-8 by 10 and 12%, respectively (*p* = 0.0274 and 0.0230). Since fraction 1 of enoxaparin showed the maximum inhibition of tested ILs, it was selected for further analytical and bio-analytical investigations.

### Concentration-dependent Effect of Fraction 1 on IL-6 and IL-8 Release

**Fig 5:**
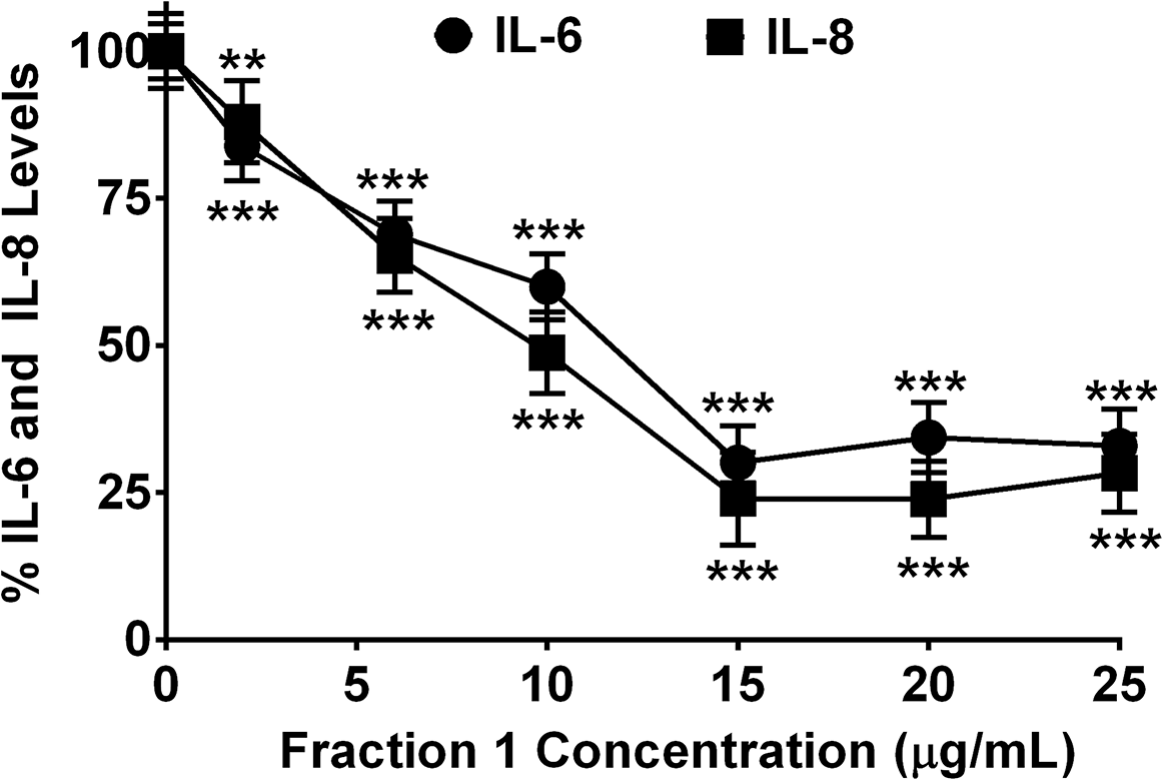
Concentration-dependent effect of IC-derived fraction 1 on interleukin release. Inhibition of IL-6 and IL-8 release by fraction 1 of enoxaparin (0 to 25 μg/mL) after *in-vitro* stimulation of epithelial cells via trypsin. Data is presented as percentage of trypsin-stimulated control (mean ± SD). **p<0.01 and ***p<0.001 versus trypsin-stimulated control.

**Fig 6:**
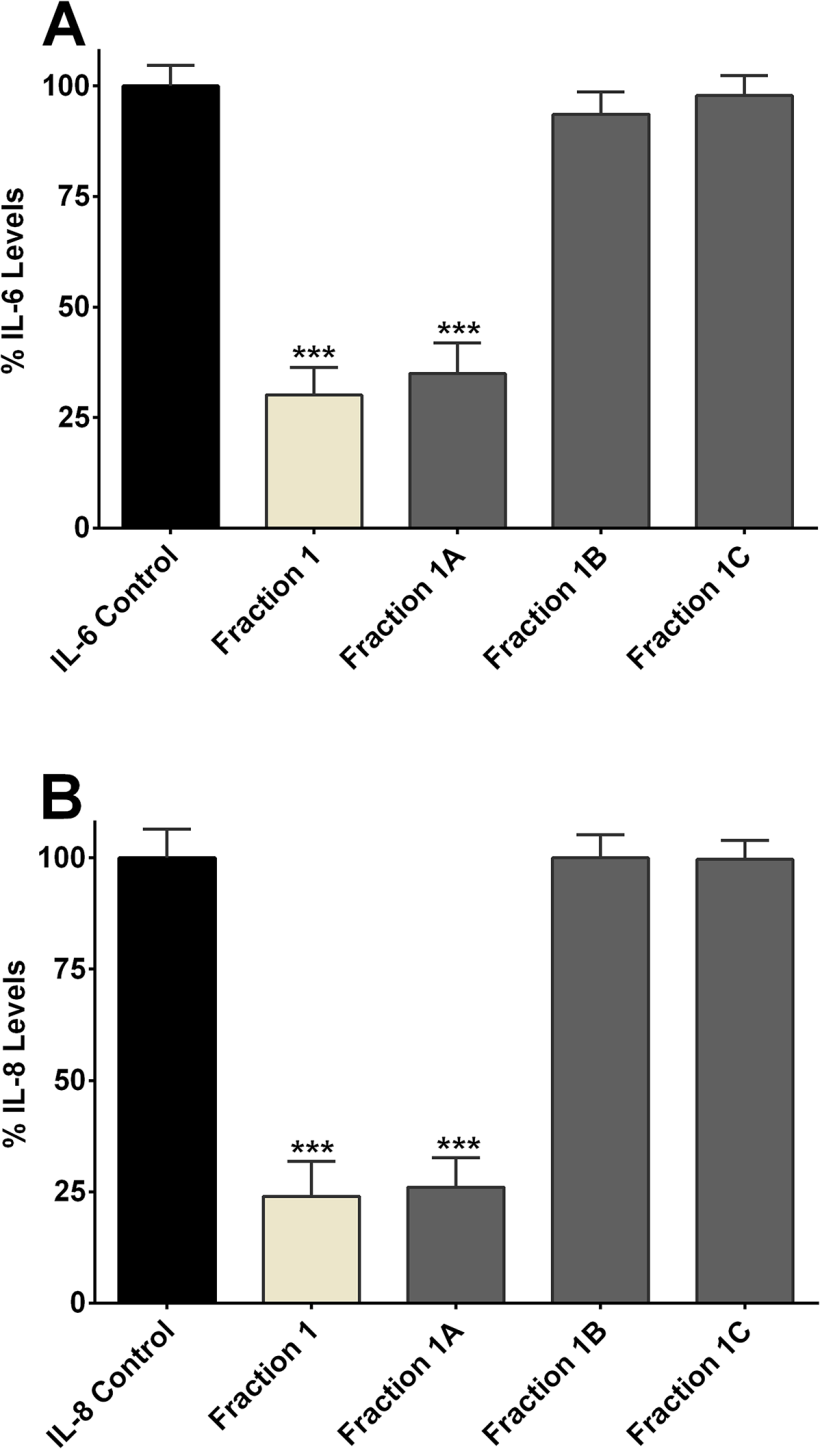
Effect of sub-fractions of IC-derived fraction 1 on interleukin release. Inhibition of IL-6 **(A)** and IL-8 **(B)** release by enoxaparin fraction 1 and its three different sub-fractions (1A, 1B and 1C) after trypsin-induced *in-vitro* stimulation of epithelial cells. Data is presented as percentage (mean ± SD) of the maximal observed IL-6 and IL-8 concentrations. ***p<0.001 versus trypsin-stimulated control.

Like enoxaparin, fraction 1 also inhibited the release of IL-6 and IL-8 in a concentration-dependent manner (Fig 5A). The maximum inhibition of IL-6 and IL-8 was observed at 15 μg/mL and higher concentrations did not result in greater inhibition. At 15 μg/mL, it inhibited the release of IL-6 and IL-8 by 70 and 76%, respectively (*p*<0.0001). Fraction 1 of enoxaparin, as can be seen in Fig 4A, is composed of three different sub-fractions eluting at 30.0, 31.2 and 32.3 minutes, respectively. Although the molecular weights of the three sub-fractions (fraction 1A, 1B and 1C) were found to be similar, the IC technique was efficient enough to separate them from each other. The separation of IC is based on the interaction of the negatively charged groups (e.g. sulfate groups) of enoxaparin fractions with the positively charged stationary phase of the separation column. The fractions with more negative charge elute later during the IC salt gradient separation. Therefore, sub-fraction 1A appears to have less negatively charged groups than fraction 1B or fraction 1C. As the potential anti-inflammatory effects of LMWHs depend on their degree, as well as pattern, of sulfation, we further investigated the effect of each sub-fraction on the release of IL-6 and IL-8. The effect of three different sub-fractions on the release of IL-6 and IL-8 is shown in Fig 6. Sub-fraction 1A inhibited the release of IL-6 and IL-8 by approximately 65% and 74%, respectively (*p*<0.0001). On the other hand, sub-fraction 1B and 1C did not significantly inhibit the release of tested ILs (*p*>0.1683). As sub-fraction 1A was found to be responsible for the majority of the inhibitory effect of fraction 1, its structure was characterised by NMR.

### Structural Characterisation of Sub-fraction 1A

**Fig 7:**
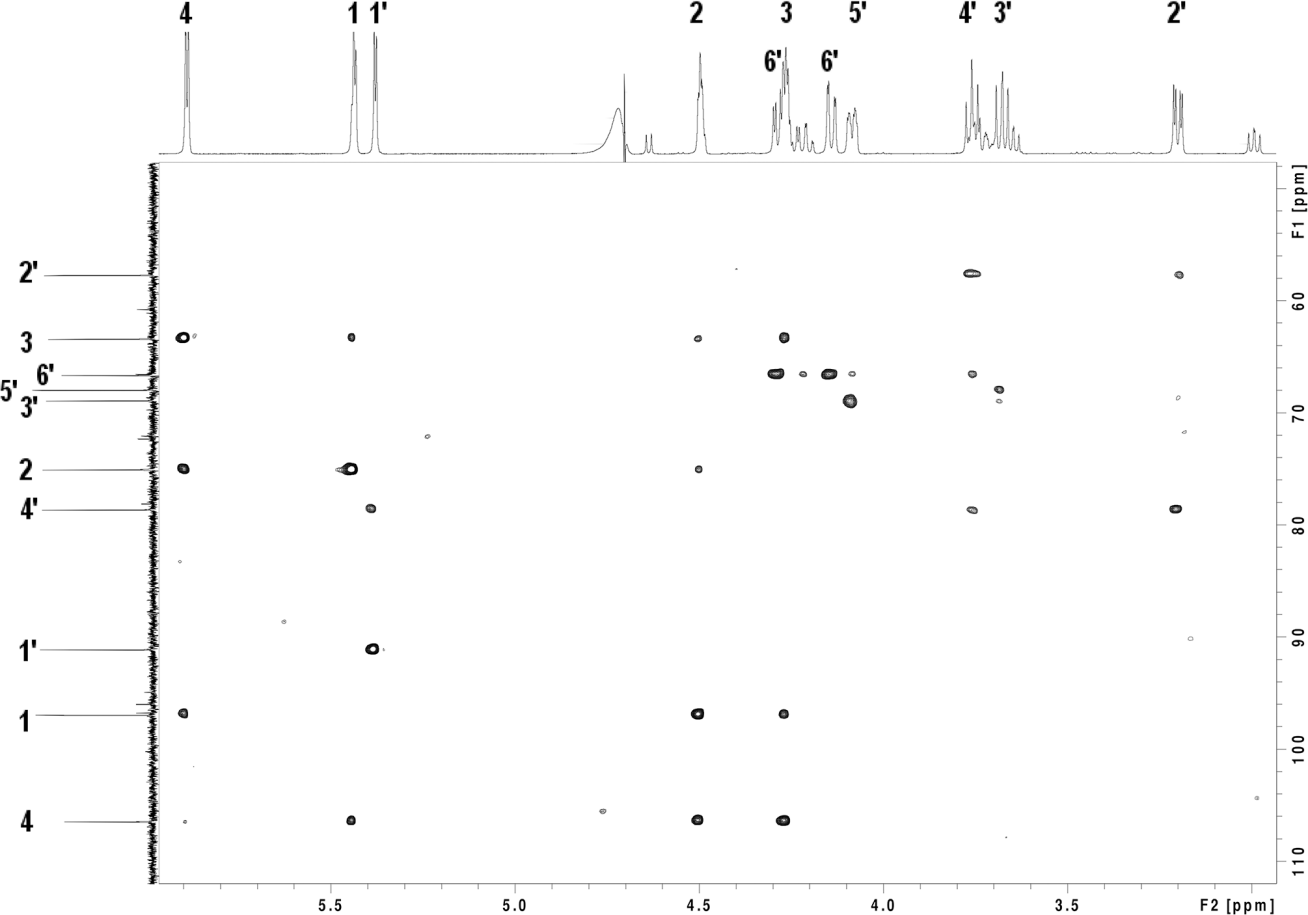
^13^C-^1^H HSQC multiplicity edited spectrum for enoxaparin sub-fraction 1A. Axis units are chemical shifts in parts per million (ppm). The dark blue contours denote correlations between carbon atoms with one or three attached protons and cyan contours carbons with a single attached proton. The atom nomenclature from Table 1 is applied to the proton and carbon axis projections.

**Fig 8:**
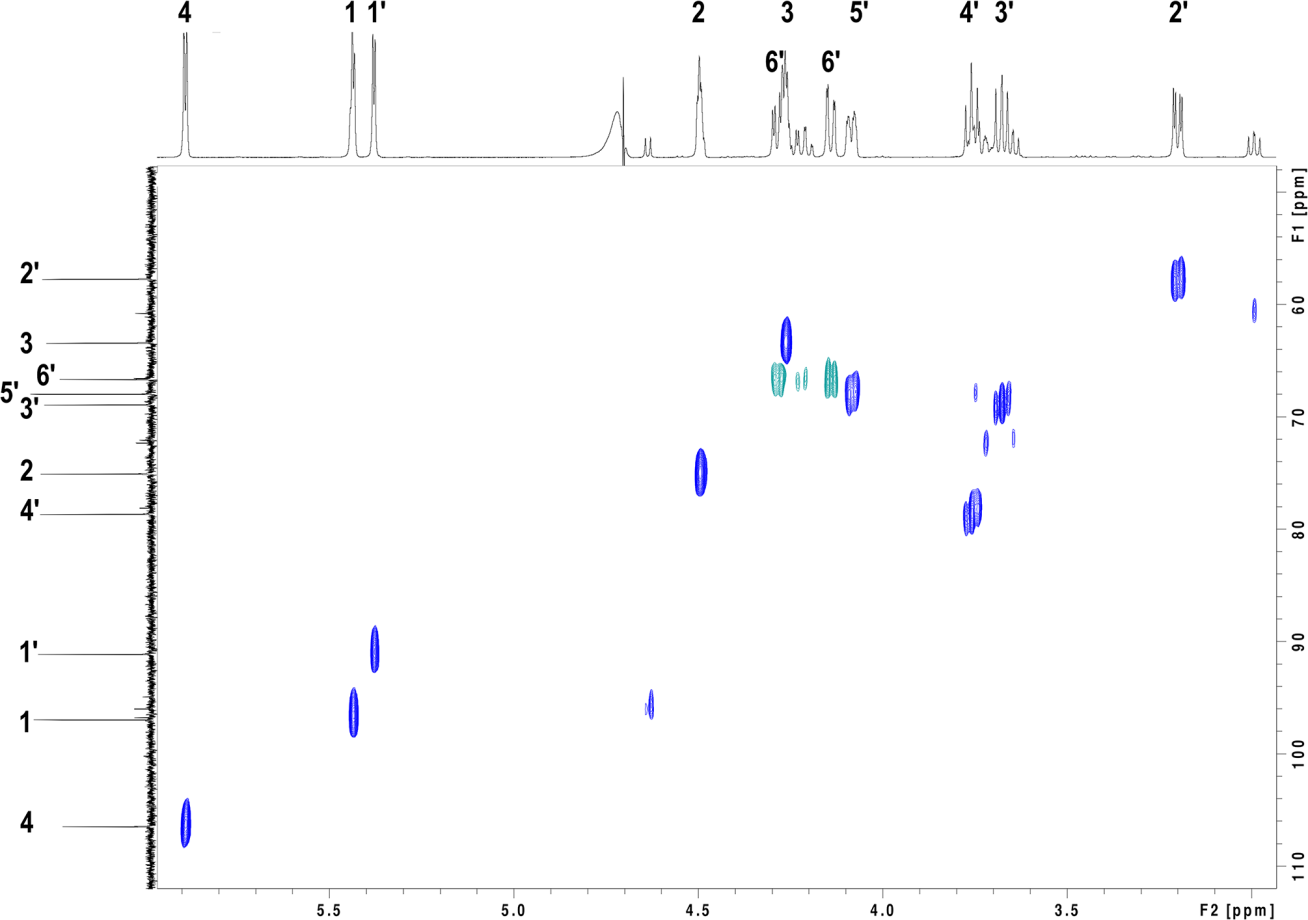
^13^C-^1^H HSQC-TOCSY 120ms spectrum for enoxaparin sub-fraction 1A. Axis units are chemical shifts in parts per million (ppm). The atom nomenclature from Table 1 is applied to the proton and carbon axis projections.

Full assignment of the dominant proton and carbon signals in the enoxaparin sub-fraction 1A was made from analysis of the NMR data. Proton connectivity networks in the individual sugar rings were established from correlations in ^1^H-^1^H TOCSY spectra. Carbon assignments were derived through one-bond correlation of these protons to carbons in the ^13^C-^1^H HSQC spectrum (Fig 7). The ^13^C-^1^H HSQC-TOCSY experiment (Fig 8) was used to discriminate between groups of interconnected protons in separate sugars for which overlap in the ^1^H-^1^H-TOCSY did not allow unambiguous assignment. The link between the two sugars was confirmed by a correlation across the glycosidic linkage between H4’ and C1 observed in heteronuclear multiple-bond correlation (HMBC) spectrum. The NMR assignments and measured J-couplings of sub-fraction 1A are summarised in Table 1, with atom numbering consistent with previously reported data [39]. It was found to be a disaccharide of enoxaparin containing a unit of α-L iduronic acid and one of α-D glucosamine-6-sulfate. The glucosamine unit of the disaccharide was present in both α and β forms, and could be correlated with chemical shift and coupling observations for a similar disaccharide fragment of heparin [39] and spectra for glucosamine-6-sulfate in the Human Metabolome Database (HMDB) repository [40]. The iduronic acid unit did not display evidence of multiple conformations at the temperature used in this study. The glucosamine was predominantly in the α**-**form based on relative integral volumes between the two forms measured in the ^1^H-1D spectrum.

**Table 1:** NMR assignments of enoxaparin disaccharide.

	1	2	3	4	1'	2'	3'	4'	5'	6'
^1^H ppm	5.435	4.496	4.263	5.89	5.379	3.192	3.673	3.754	4.084	4.141 4.285
^3^J_HH_ Hz	3.36	3	3	4.44	3.48	10.16 3.57	10.43	9.39	9.98 3.57	11.26 3.8
^13^C ppm	96.61	75.05	63.41	106.3	90.94	57.71	68.94	78.39	67.99	66.7

Atoms in the glucosamine unit are denoted by the prime superscript.

Cq (COO-) 169.1 ppm, C6 (Ido) 144.79 ppm.

### Anti-factor Xa Analysis of Disaccharide of Enoxaparin

**Fig 9:**
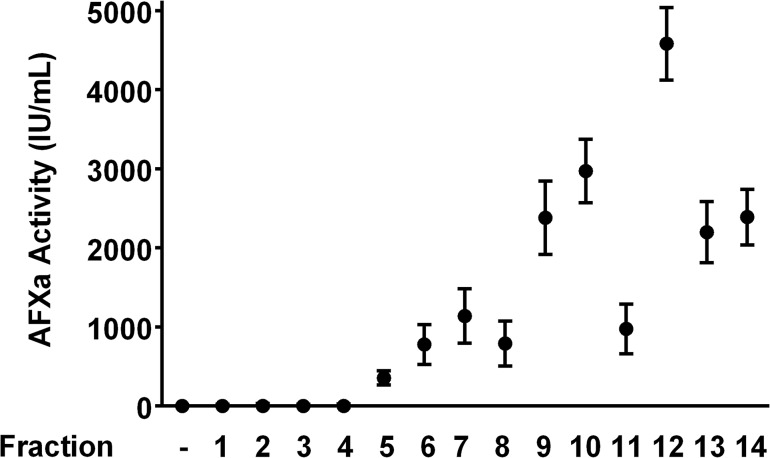
Anti-factor Xa activity of fourteen enoxaparin fractions derived using IC technique. The anti-factor Xa assay is described in the experimental section. Data is presented as mean ± SD (n = 3).

The anti-factor Xa activity of fourteen IC-derived fractions of enoxaparin is shown in Fig 9. As expected, the disaccharide of enoxaparin did not show any anti-factor Xa activity. Inhibition of factor Xa by anti-thrombin requires a formation of a complex composed of factor Xa, anti-thrombin and enoxaparin [41]. To mediate an interaction between factor Xa and anti-thrombin, a minimum chain length of 5 saccharide (pentasaccharide) sequence of enoxaparin is required. It means the pentasaccharide of enoxaparin is the smallest fraction required to mediate inactivation of factor Xa by anti-thrombin. The identified disaccharide of enoxaparin was too small to act as a template through which anti-thrombin mediated inactivation of factor Xa can occur. This finding is significant because the risk of bleeding is increased when enoxaparin is used for medical conditions other than where an anticoagulation effect is required. The identified disaccharide lacked the anticoagulant activity and therefore can eliminate the potential risk of bleeding associated with enoxaparin.

### Effect of Desulfated Disaccharide of Enoxaparin on IL-6 Release

**Fig 10:**
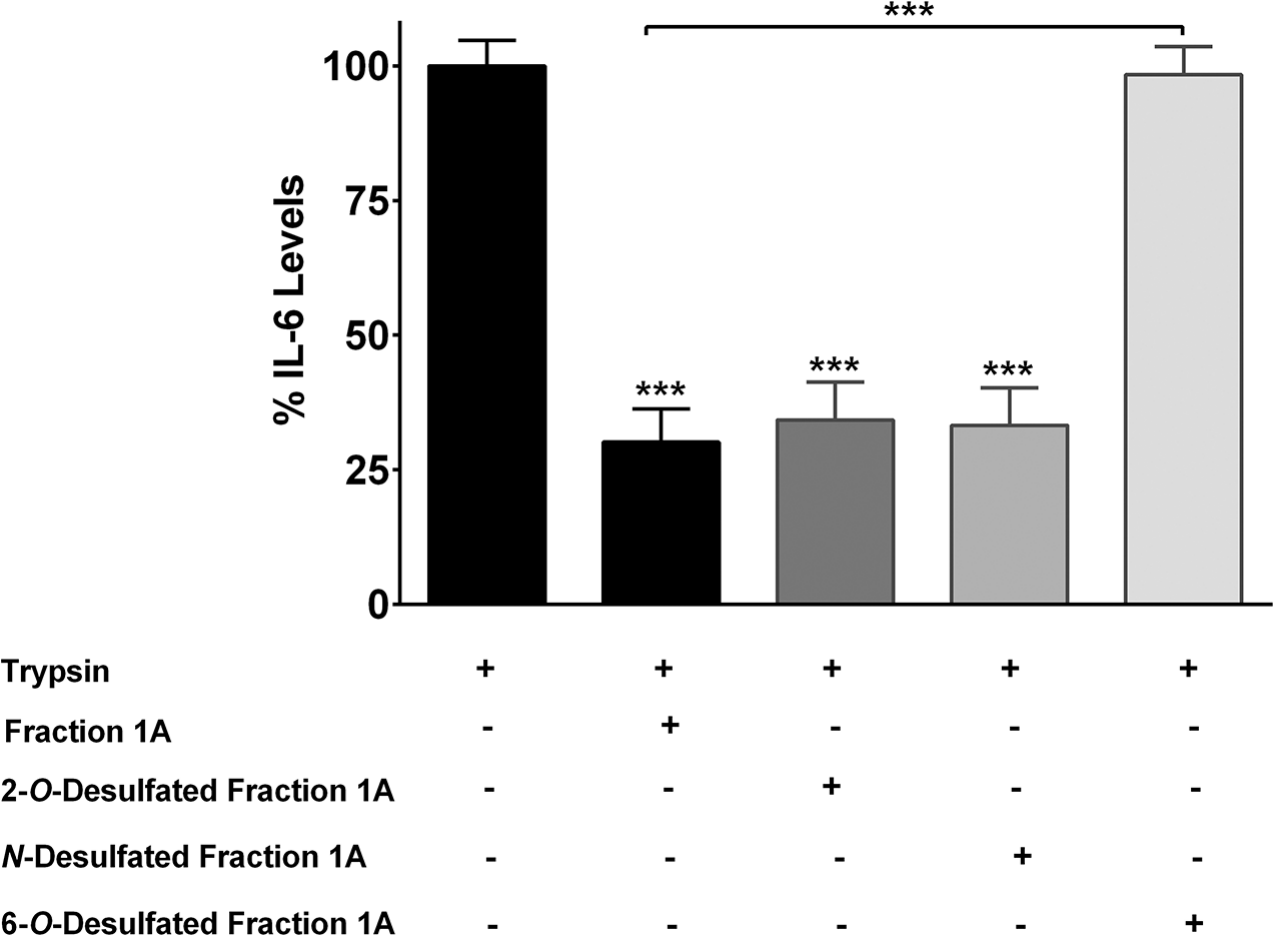
Effect of various desulfated fraction 1A on IL-6 release. Epithelial cells were stimulated with trypsin in the presence of either 2-*O*-desulfated, *N*-desulfated or 6-*O*-desulfated fraction 1A of enoxaparin. Data is presented as mean ± SD. ***p<0.001 versus trypsin-stimulated control.

Heparins and its LMW derivatives are highly sulfated linear molecules composed of alternating D-glucosamine residues linked 1→4 to either L-iduronic acid or D-glucuronic acid. The principle unit in heparin disaccharide is trisulfated and represents approximately 75 to 90% of the heparin chain [42]. Heparin chains are sulfated at the 2-*O* position of iduronate residues, glucosamine residues and glucuronic acid; 6-*O* position of glucosamine residues and 3-*O* position of glucosamine residues [43]. The presence and location of specific sulfate groups is critical to elicit various biological activities of heparins [20]. Therefore, the selectively desulfated disaccharide was tested for its ability to inhibit the release of IL-6 (Fig 10). As expected, the disaccharide inhibited the release of IL-6 by approximately 70% (*p*<0.0001). While 2-*O* and *N*-desulfated disaccharide did not significantly change the observed inhibition of disaccharide (*p*>0.6018), the 6-*O* desulfated disaccharide-induced inhibition of IL-6 was reduced by 98% (*p*<0.0001).

The importance of *O-* or *N*-sulfate groups in controlling various biological activities of heparins has been reported before. For instance, the presence of 3-*O*-sulfate groups is found to be critical for retaining heparin’s anticoagulant activity [43]. On the other hand, 2-*O* and *N*-sulfate groups are reported to be essential for its pro-inflammatory activity mediated through chemokine-induced chemotaxis [44,45]. *N*-sulfate groups are also reported to be crucial for proliferation of airway smooth muscle cells [46], cytokine-mediated inflammation [47] and integrin-mediated leukocyte adhesion [48]. Similarly, the importance of 6-*O*-sulfate groups in controlling the anti-inflammatory activity of heparins has been reported before. For instance, the presence of sulfate groups at 6-*O*-position was found to be critical for: i) selectin-mediated leukocyte adhesion [49]; ii) chemokine-mediated adhesion and transmigration [50] and iii) binding to various receptors, such as fibroblast growth factor receptors [51,52]. The present study, for the first time, reports the requirement of 6-*O* sulfate groups for the inhibition of IL-6 and IL-8 release from human pulmonary epithelial cells.

### Binding of Proteins to Disaccharide of Enoxaparin

Decreased levels of IL-6 and IL-8 observed through ELISA could potentially be because of: i) direct binding of disaccharides to trypsin and, hence, inhibiting the trypsin-induced activation of PAR (lung epithelial cell receptors); ii) binding of disaccharides to IL-6 and IL-8 and, hence, their reduced detection through ELISA or iii) binding of disaccharides to PAR and, hence, inhibiting PAR-induced release of IL-6 and IL-8 through specific intracellular signalling pathways. Specific binding between glycominoglycans (e.g. enoxaparin) and proteolytic enzymes (e.g. thrombin) has been reported before [53]. Direct binding of glycosaminoglycan disaccharide and proteolytic trypsin could impair the ability of trypsin to bind and activate the PAR. Therefore, we needed to confirm that the observed inhibition of release of ILs was not due to this potential *in-vitro* artefact of our experimental cell system. Hence, HP-SEC was used to investigate the putative binding between disaccharide and trypsin. In the current HP-SEC method, two separation columns were connected in series. The first column (Superdex peptide 10/300 GL) allowed the separation of proteins and carbohydrates having low MWs (0.1 to 8 kDa) and the second column (Superdex 75 10/300 GL) provided the effective separation of analytes with high MWs (3 to 70 kDa). Moreover, the detection of the tested analytes was carried out using a charged aerosol detector (CAD) instead of ultra-violet (UV) detector. The advantage of CAD, as compared to UV detection, is that the peak areas of analytes are not dependent on a UV chromophore, for which extinction coefficients can vary by orders of magnitude depending on the chemistry of analyte, but are dependent on the mass of analytes. The performance of the HP-SEC method was determined using intra- and inter-day precision and accuracy, and the inter-day retention time of each analyte. The intra- and inter-day precision relative standard deviation (RSD) for each analyte peak was less than 4.1% (n = 6) and 5.3% (n = 5), respectively. The intra- and inter-day accuracy RSD for each analyte peak was less than 4.3% and 5.9%, respectively. Inter-day retention time RSD for each of the analyte peaks were less than 0.7% (n = 6).

**Fig 11:**
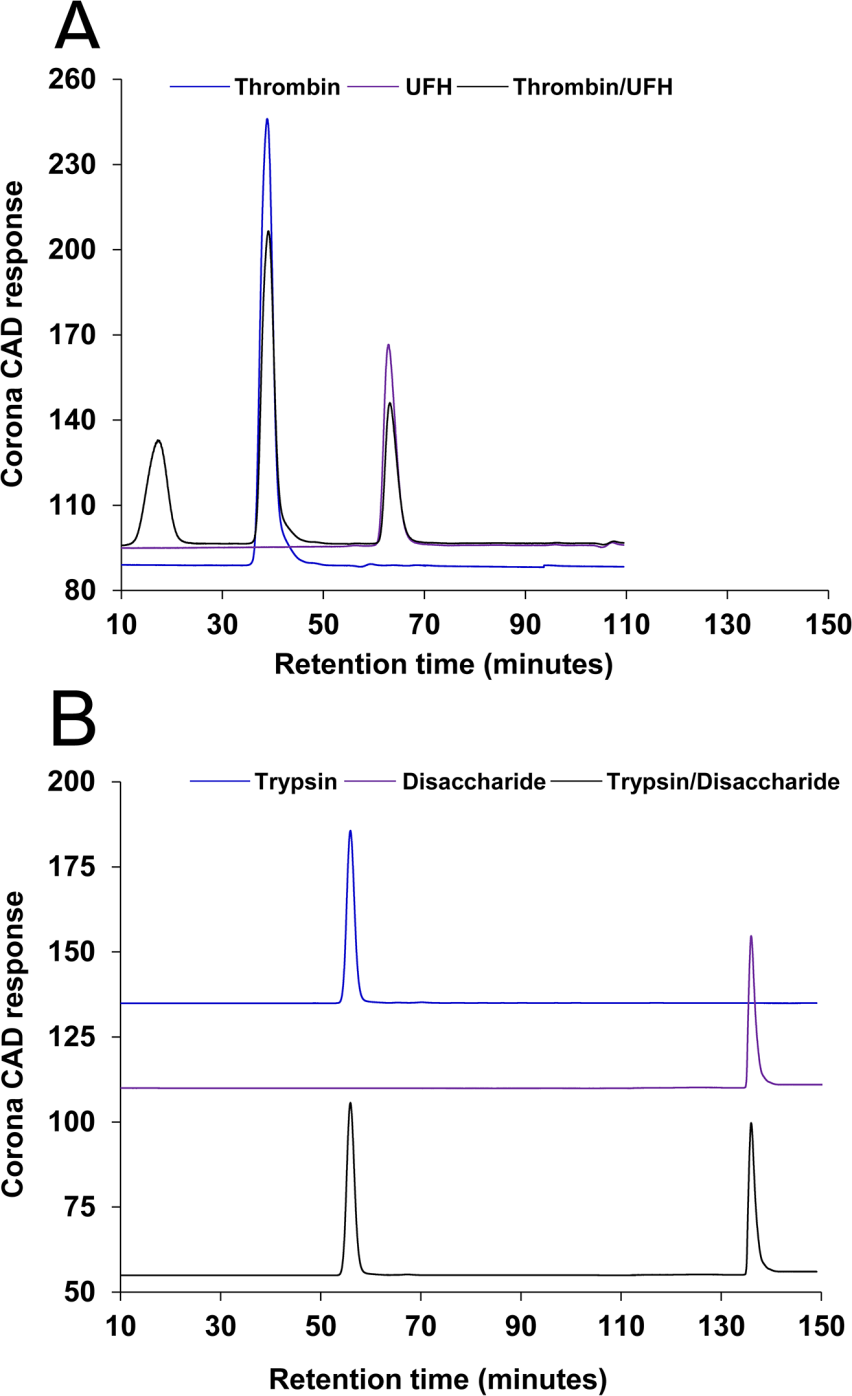
Binding of proteins with disaccharide of enoxaparin. Interaction of UFH with thrombin. HP-SEC chromatograms of UFH (10 μM), thrombin (10 μM) and a mixture of UFH/thrombin (1:1 molar ratio) **(A)**. Interaction of IC-derived disaccharide with trypsin. HP-SEC chromatograms of enoxaparin disaccharide (500 μM), trypsin (10 μM) and a mixture of trypsin/disaccharide (1:50 molar ratio) **(B)**. Details of the HP-SEC experimental conditions are given in Methods.

The ability of HP-SEC to determine the binding between glycominoglycans and proteins was first investigated by analysing UFH (10 μM) and thrombin (10 μM). The HP-SEC chromatograms of UFH, thrombin and a mixture of UFH/thrombin (1:1 molar ratio) are shown in Fig 11A. UFH and thrombin peaks were eluted at 65 and 37 minutes. In HP-SEC analysis, the separation of analytes is based on their molecular size and, therefore, thrombin with a MW of 37 kDa eluted earlier than UFH (MW 15 kD). However, when a mixture containing UFH and thrombin was analysed (Fig 11A), a new peak was eluted at 16 minutes, suggesting the observed component had a higher MW compared to thrombin or UFH. When glycosaminoglycans bind to proteins, it results in the formation of a high MW complex. Therefore, the observed reduction in the peak areas of thrombin and UFH, and simultaneous elution of a new peak indicated the binding between UFH and thrombin. UFH is a mixture of complicated oligosaccharides ranging from disaccharides to oligosaccharides larger than 50–100 saccharides. Only oligosaccharides with a minimum of 18 saccharides can bind to thrombin and, therefore, as can be seen in Fig 11A, approximately 25% of UFH oligosaccharides did not bind to thrombin. The disaccharide of enoxaparin and trypsin was then analysed by HP-SEC. As shown in Fig 11B, trypsin and disaccharide were eluted at 53 and 136 minutes respectively. There was no difference in the chromatographic profile of disaccharide or trypsin when a mixture of trypsin/disaccharide (1:50 molar ratio) was injected into the HP-SEC system (Fig 11B). For example, the peak area of trypsin remained unchanged when a solution containing either trypsin or mixture of trypsin and disaccharide was injected. Also, no extra peak was observed when a mixture was analysed suggesting the decreased levels of ILs were not because of the interaction between disaccharide and trypsin. Apart from proteolytic enzymes, glycosaminoglycans are shown to interact with cytokines, chemokines and growth factors. For example, heparins can bind to IL-10, MCP-1 and epidermal growth factors and then modulate their biological activity [54–56]. However the HP-SEC results indicated that the disaccharide of enoxaparin did not bind to either IL-6 or IL-8.

Trypsin is known to activate PAR by cleaving the receptor’s extracellular N-terminal domain. This cleavage results in the formation of a new N-terminus which then interacts with trypsin causing activation of G-protein-coupled signal transduction pathway [34]. Interestingly, the N-terminal domain of various proteins is reported to be a binding site for heparins as well [57,58]. For example, heparins bind to the N-terminal domain of serine protease inhibitor resulting in the anticoagulant effect of enoxaparin [59]. Since, disaccharide of enoxaparin did not interact with either trypsin or the tested ILs, the observed decrease in IL levels could potentially be due to the interaction between N-terminal domain of PAR and disaccharide. Future experiments should be aimed to provide direct evidences of interactions between disaccharide of enoxaparin and N-terminus of PAR as well as the mechanistic insights via which the potential interaction occurs. In the current study only a single type of epithelial cell line was tested to investigate the effect of enoxaparin on the release of IL-6 and IL-8. However, upon stimulation, other types of epithelial cell lines with potentially different types of cell surface receptors also release pro-inflammatory mediators. Therefore, it would be interesting to investigate the effects of enoxaparin on such types of epithelial cells.

In summary, the identified fraction responsible for the anti-inflammatory effect of enoxaparin is composed of two saccharides. Therefore, it avoids the risk of bleeding as a minimum of pentasaccharide sequence is required for the anticoagulant effect. As IL-6 and IL-8 are important pro-inflammatory mediators involved in the early pathogenesis of asthma, inhibition of their release, as seen with the identified disaccharide, could provide a much needed novel therapeutic option for the management of clinical manifestations associated with this medical condition. However, future clinical studies should be conducted to confirm the preliminary findings of the current *in-vitro* study.
